# In Vitro Characterization of the Innate Immune Pathways Engaged by Live and Inactivated Tick-Borne Encephalitis Virus

**DOI:** 10.3390/vaccines9060664

**Published:** 2021-06-17

**Authors:** Aurora Signorazzi, Jeroen L. A. Pennings, Marilena P. Etna, Malou Noya, Eliana M. Coccia, Anke Huckriede

**Affiliations:** 1 Department of Medical Microbiology & Infection Prevention, University Medical Center Groningen, University of Groningen, 9713GZ Groningen, The Netherlands; a.signorazzi@umcg.nl (A.S.); malou.noya@live.nl (M.N.); 2 Centre for Health Protection, National Institute for Public Health and the Environment (RIVM), 3721MA Bilthoven, The Netherlands; jeroen.pennings@rivm.nl; 3 Department of Infectious Diseases, Istituto Superiore di Sanità, 000161 Rome, Italy; marilenapaola.etna@iss.it (M.P.E.); eliana.coccia@iss.it (E.M.C.)

**Keywords:** tick-borne encephalitis virus, TBE vaccine, peripheral blood mononuclear cells, RNA sequencing, interferon, RIG-I, TLR

## Abstract

Tick-borne encephalitis virus (TBEV) infection can lead to inflammation of the central nervous system. The disease can be effectively prevented by whole inactivated virus vaccines. Here, we investigated the innate immune profile induced in vitro by the antigen component of the vaccines, inactivated TBEV (I-TBEV), to gain insights into the mechanism of action of the TBE vaccine as compared to the live virus. To this end, we exposed human peripheral blood mononuclear cells (PBMCs) to inactivated and live TBEV and assessed cellular responses by RNA sequencing. Both inactivated and live TBEV significantly induced an interferon-dominated gene signature and an increased RIG-I-like receptor (RLR) expression. Using pathway-specific inhibitors, we assessed the involvement of pattern recognition receptors in the sensing of inactivated or live TBEV. Only RLR pathway inhibition significantly suppressed the downstream cascade induced by I-TBEV, while responses to the replicating virus were impacted by the inhibition of RIG-I-like, as well as Toll-like, receptors. Our results show that inactivated and live TBEV predominantly engaged an interferon response in our in vitro PBMC platform, and indicate RLRs as the main pattern recognition receptors involved in I-TBEV sensing.

## 1. Introduction

Tick-borne encephalitis is an inflammation of the central nervous system caused by the tick-borne encephalitis virus (TBEV), a flavivirus endemic in parts of Europe and Asia [1]. The virus is responsible for thousands of cases of human encephalitis every year, and its incidence in Western Europe has been growing in the past decades [2,3]. Currently there are no specific treatments for TBE, but inactivated virus vaccines containing aluminum hydroxide as adjuvant are available and effectively prevent infection also from heterologous TBEV strains [4,5]. The use of TBE vaccines has led to successful containment of the disease in countries with high immunization coverage [6].

The development of adaptive responses evoked by the TBE vaccine has been investigated in detail, showing the induction of envelope protein-directed antibodies and of TBEV-specific CD4^+^ T cells [7]. However, a full picture of the innate immune responses induced by the vaccine is yet to be presented [8]. A better insight into the molecular pathways activated by the TBE vaccine could shed light on the mechanisms involved in the generation of vaccine-conferred protection. Indeed, the successful induction of innate immune responses has repeatedly been shown to correlate with the potency of several vaccines [9,10,11,12]. As the TBE vaccine has been proven to have a high field effectiveness [13], the investigation of the specific pathways it induces could help delineate vaccine-induced innate immune functions associated with a favorable vaccination outcome.

Several studies have highlighted how specific innate immune pathways activated by certain vaccine components, such as viral RNA, can determine the efficacy of influenza and other vaccines [14,15,16]. Here, we aimed to delineate pattern recognition receptors (PRRs) and immune signaling pathways engaged by the TBE vaccine, in particular by inactivated TBEV, the main component of the vaccine. To this end, we employed an in vitro system consisting of human peripheral blood mononuclear cells (PBMCs) which we had developed to assess the conformity of different TBE vaccine batches [17]. We exposed the human PBMCs to I-TBEV (of high or low quality) or live TBEV and assessed the response by RNA sequencing. Our results show that TBEV (live or inactivated) induced a distinct interferon-dominated transcriptomic signature, but not a generalized inflammatory response. Using pathway-specific inhibitors and reporter cell lines, we showed that both I-TBEV and live TBEV activated the cells through engagement of RIG-I-like receptors (RLRs), but only live TBEV was also able to trigger selected Toll-like receptors (TLRs) in reporter cell lines and frozen-thawed PBMCS.

This study brings new insights into the innate response elicited and the signaling pathways engaged in vitro by live TBEV and by the inactivated virus, the primary constituent of the TBE vaccine.

## 2. Materials and Methods

### 2.1. Vaccine and Virus

*Vaccine formulations*. Formalin-inactivated TBEV of the K23 strain (I-TBEV; 60 μg/mL protein) was kindly provided by GlaxoSmithKline (GSK, Marburg, Germany). I-TBEV, the antigen-containing fraction of the Encepur vaccine, consists of whole, formalin-inactivated TBEV in a 42% sucrose solution and thus contains virions including structural proteins and viral genomes. To produce non-conforming batches, a strategy that reduces binding by TBEV-specific antibodies [18] was followed: I-TBEV was heat-treated at 42 °C for 4 weeks in glass vials (HT I-TBEV). A 42% low-endotoxin sucrose (Sigma-Aldrich, St. Louis, MO, USA) solution in DMEM medium (Gibco, Life Technologies; Paisley, UK) was used as control (matrix), as per indications of GSK.

*Culture and quantification of TBEV.* Live TBEV (European strain Neudörfl) was obtained from the European Virus Archive (Marseille, France). The European strain K23 used for the GSK vaccine could unfortunately not be obtained; however, nucleotide and protein blasting of the two strains revealed a 97% and 99% identity, respectively. The virus seed was expanded on Vero E6 cells (ATCC, Rockville, MD, USA) as previously described [17]. The infectious particles in the supernatant were quantified by a plaque assay on A549 cells (ATCC), which are highly susceptible to the virus cytopathic effect [19]. Briefly, monolayers of A549 cells cultured in 12-well culture plates were inoculated with 10-fold dilutions of TBEV-containing supernatants for 4 h at 37 °C. The cells were overlaid with 2% agarose in 2× MEM medium and were incubated for 4 days at 37 °C with 5% CO_2_. The cells were then fixed with 10% formaldehyde for 1 h and the overlay was discarded and the cells stained with crystal violet to visualize the plaques. The virus titers were expressed as plaque-forming units (PFU) per mL.

### 2.2. Cells

*PBMCs*. Buffy coats were purchased from the Dutch blood bank (Sanquin, Groningen, The Netherlands) who had obtained consent of the donors to use the cells for scientific research. It should be noted that the TBE vaccination status of the donors in this study was unknown; however, given the absence of a governmental recommendation and the very low incidence of TBE in The Netherlands (with a total of only 12 cases reported so far), it is highly unlikely that the donors had been previously exposed to the virus or the vaccine [20]. Peripheral blood mononuclear cells were isolated as previously described [21]. Briefly, buffy coats were mixed with RPMI-1640 (Gibco, Life Technologies; Paisley, UK) and were layered on Ficoll Paque (GE Healthcare, Uppsala, Sweden). After centrifugation, PBMC fractions were collected and red blood cells were lysed with Ammonium-Chloride-Potassium (ACK) lysis buffer (ThermoFisher Scientific, Waltham, USA). PBMCs were then stored in cryopreservation medium (90% FCS, 10% DMSO) in liquid nitrogen until needed. For the experiments, PBMCs were thawed as previously described [21], and seeded at a density of 2 × 10^6^ cells/mL in 24-well plates in RPMI-1640 supplemented with 10% fetal calf serum (FCS; Life Science Production, Bedford, UK), 50 µM β-mercaptoethanol, and 1% penicillin/streptomycin (all from Gibco). Cells were incubated at 37 °C, 5% CO_2_.

*Reporter cells.* HEK-Blue™ cells (InvivoGen, Toulouse, France) co-express PRRs and an NF-kB-inducible secreted embryonic alkaline phosphatase (SEAP) reporter gene that can be monitored using the detection medium QUANTI-Blue™. Human HEK-Blue™ TLR2, TLR3, TLR4, TLR5, TLR7, TLR8, TLR9, and NOD2 cells (and parental Null cells) were cultured at 37 °C in 5% CO_2_ according to the manufacturer’s instructions and 50.000 cells/well were plated in a 96-well plate and stimulated. After 48 h of incubation, 50 µL of supernatant were added to 150 µL of QUANTI-Blue™. After 30 min of incubation at 37 °C, the plates were read in an ELISA reader (630 nm). The results are expressed as relative activation of cells in comparison to the activation level obtained upon stimulation with 2.5 μg/mL of TNF-α (ProsPec, Rehovot, Israel), which was set as 100%.

### 2.3. Cell Stimulation

PBMCs and reporter cell lines were stimulated for 24 or 48 h with I-TBEV, HT I-TBEV (or the matrix control) at dilutions from 1:4000 to 1:16 (equivalent to antigen concentrations from 0.015–4 μg/mL). Incubation with live TBEV was performed for 24 or 48 h at a multiplicity of infection (MOI) from 1 to 100.

*Inhibitors.* Amlexanox and BX795, inhibitors for TBK1/IKKε (kinases involved in the RLR pathway), and Pepinh-MYD, an inhibitor peptide for MyD88 (signal transducer for TLR pathways), were used to pre-treat the cells for 1 or 6 h at 37 °C in 5% CO_2_ before subsequent stimulation with I-TBEV or positive controls. All inhibitors were purchased from Invitrogen, and were used according to the manufacturer’s specifications at a concentration of 5–50 μg/mL (Amlexanox), 0.1–2 μM (BX795) and 5–50 μM (Pepinh-MYD).

*Positive controls.* TLR7/8 ligand R848 (10 μg/mL) and poly I:C-HMW/LyoVec (0.5 μg/mL) (both from Invitrogen) were used as controls to assess the stimulation of the cell platforms.

### 2.4. Cell Lysis and RNA Isolation

To detect changes in the gene expression of stimulated cells, cell lysates of PBMCs were collected and the mRNA was isolated for subsequent analysis by RT-qPCR or RNA sequencing. Cells were lysed by adding 350 μL RLT buffer (Qiagen, Hilden, Germany) + 1% β-mercaptoethanol. The lysates were then stored at −20 °C until further analysis. RNA isolation from the lysates was performed using the RNeasy Mini Kit (Qiagen) following the instructions of the manufacturer.

### 2.5. RT-qPCR

cDNA from the isolated RNA was generated using the Primescript RT Reagent kit (Takara, Saint-Germain-en-Laye, France) according to the manufacturer’s instructions. The cDNA was then analyzed by qPCR: the reaction (10 μL 2× ABsolute qPCR SYBR^®^ Green Mix (ThermoFisher Scientific), 1 μL 10 mM forward primer, 1 μL 10 mM reverse primer, 1.5 μL cDNA and 6.5 μL H_2_O) was carried out for 10 min at 95 °C, 40 cycles of 15 s at 95 °C and 1 min at 60 °C in a CFX96 Touch Real-Time PCR Detection System (Biorad, Hercules, CA, USA). The gene expression levels of the target genes were normalized against the housekeeping gene GAPDH and were quantified relative to the expression levels in non-treated cell cultures (primer sequences shown in Appendix A Table A1). Data were analyzed according to the comparative Ct method [22] and are expressed as fold change.

### 2.6. RNA Sequencing

*Library preparation and next generation sequencing (NGS).* All NGS experiments (performed on RNA isolated from PBMCs of a healthy donor) were conducted by QIAGEN Genomic Services, using the QIAseq UPX 3′ Transcriptome Kit. Sequence reads were mapped to the human genome (version: hg38, annotation: NCBI RefSeq GRCh38.p11) using CLC Genomics Workbench (version 12.0.4) and CLC Genomics Server (version 11.0.3). This resulted in a table with gene count data for 54.362 genes and 24 samples.

*Data analysis.* Gene count data were further analyzed in R statistical software (version 3.6.2, www.r-project.org, accessed on 1 June 2020) using the following packages: DESeq2, limma, gplots, and rgl. Genes that had zero counts in all samples were considered unexpressed and were discarded from further analysis. Gene count data were normalized using a variance stabilizing transformation (VST) on the remaining genes. Differentially expressed genes (DEGs) were selected by one-way ANOVA. *p*-values were corrected for multiple testing using the Benjamini–Hochberg False Discovery Rate (FDR). Genes were considered differentially expressed if they had an FDR ≤ 5% and a Fold Change (FC) ≥ 2 (determined as the maximum vs. minimum average group value across the compared groups). Differences in gene expression compared to the control group were visualized by a heatmap combined with hierarchical clustering (using Euclidean distance and Ward.D linkage) as well as by Principal Component Analysis (PCA). Functional annotation and over-representation analysis of DEGs was carried out by using DAVID [23]. Additional functional analyses were conducted using the software packages Cytoscape plug-in ClueGO [24,25], Ingenuity Pathway Analysis (QIAGEN) and Reactome [26].

### 2.7. Quantification of Cytokines and Chemokines

Culture supernatants were harvested after 24 h of PBMC stimulation with I-TBEV, HT I-TBEV, matrix (all diluted at 1:250 v/v) and live TBEV (MOI of 10) and stored at −80 °C. The production of CXCL-10, MCP-1, and IL-8 was quantified by Cytometric Bead Assay (BD Biosciences, San Diego, CA, USA) according to manufacturer instructions.

### 2.8. Statistical Analysis

Statistically significant differences across groups in qPCR, cytokines and HEK Blue cell analyses were determined using two-way ANOVA followed by Tukey’s post-hoc test for multiple comparisons. A *p*-value of *p* < 0.05 was considered significant and indicated by *; ** stand for 0.01 and *** for 0.001. Statistical analyses were performed with GraphPad Prism version 8.0 (GraphPad Software, San Diego, CA, USA).

## 3. Results

### 3.1. RNA-Seq Identifies an I-TBEV-Specific IFN-Dominated Signature in Human PBMCs

To investigate the molecular factors and pathways involved in the human innate response to the antigen component of the TBE vaccine, we analyzed the transcriptional profile induced by I-TBEV in PBMCs from a healthy donor. The PBMCs, isolated and kept in liquid nitrogen until use, were thawed and stimulated for 24 h—unless otherwise specified—with high quality (conforming) I-TBEV, non-conforming (heat-treated) I-TBEV (HT I-TBEV), matrix or live TBEV, or were left untreated. For each treatment group, 5 replicates were included (except for the non-stimulated control group, consisting of 4 replicates), their RNA was extracted and RNA sequencing (RNA-Seq) was performed. Gene expression was quantified with sequencing reads mapped to the human genome. Variance-stabilizing normalized gene read counts were used to identify differentially expressed genes (DEGs) following treatment (see Section 2). From these data, two RNA-Seq analyses were conducted: one examining the responses in cells treated with I-TBEV (or with its negative controls HT I-TBEV and matrix, or not stimulated—Analysis 1), and one comparing the transcription profiles of I-TBEV-treated, live TBEV-treated and untreated cells (Analysis 2).

**Figure 1:**
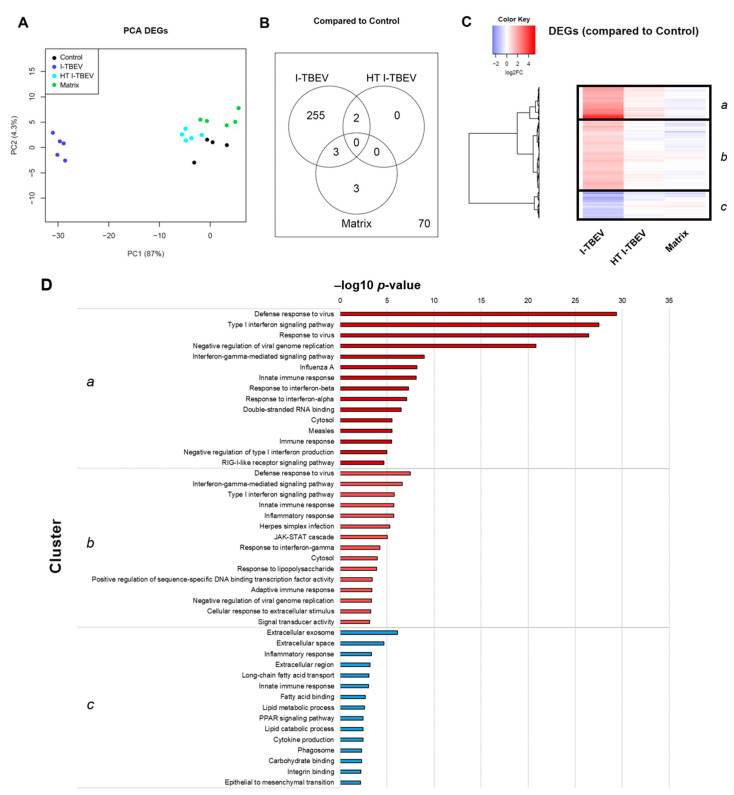
Transcriptional analysis of PBMCs stimulated with conforming and non-conforming I-TBEV (Analysis 1). PBMCs from a healthy donor were stimulated for 24 h with inactivated TBEV (I-TBEV), heat-treated I-TBEV (HT I-TBEV) or sucrose matrix at a concentration of 0.24 µg/mL (or an equivalent volume for the matrix). After treatment, the cells were lysed and processed for RNA sequencing. (**A**) Principal component analysis based on differentially expressed genes (DEGs) showing relative (dis)similarity for the samples. (**B**) Venn diagram showing DEGs with a fold change (FC) > 2 in expression between the different treatment groups and the control group. Outside the circles, the number of DEGs with a FC < 2. (**C**) Heatmap representing the fold change of DEGs in the three treatment groups, normalized to the control group (set as FC = 1). (**D**) Top 15 GO terms and KEGG pathways identified in each cluster following functional enrichment. The bar shows the –log10 of the raw *p*-value.

**Figure A1:**
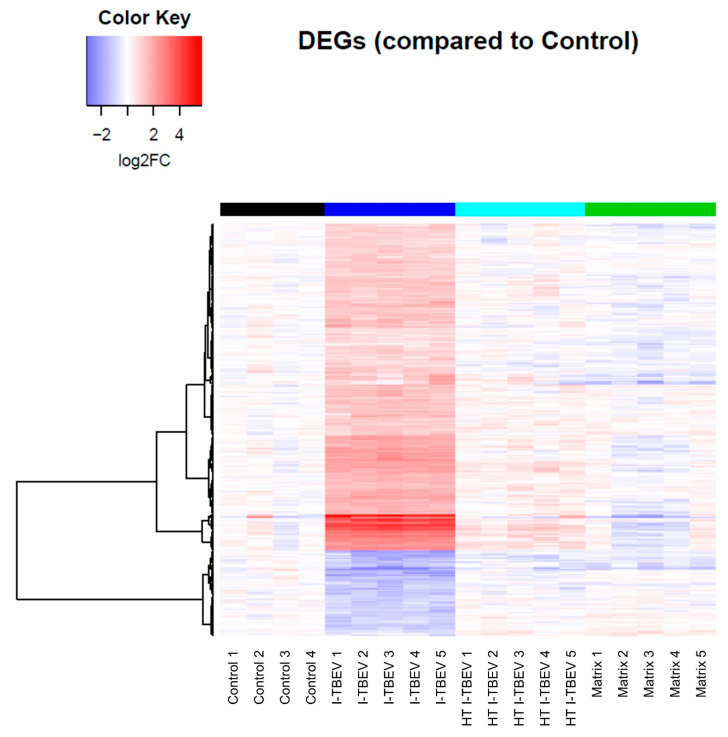
Transcriptional analysis of I-TBEV-treated PBMCs. Heatmap showing the fold change (FC) of differentially expressed genes (DEGs) in each sample, normalized to the control group.

In Analysis 1, among the four treatment groups, 333 genes were considered differentially expressed based on the specified threshold level (fold change ≥2 and false discovery rate ≤5% for the average expression across the different groups; Figure 1); 264 genes (79.3%) were found to be upregulated upon I-TBEV treatment; and 69 (20.7%) were downregulated. Principal component analysis (PCA) of differentially expressed genes (Figure 1A) showed a distinct profile for I-TBEV samples, while samples in the matrix and HT I-TBEV groups clustered closer to the non-stimulated (control) group. Upon further selection of DEGs with a strong change in expression compared to the control group (minimal fold change of 2), 255 genes were found to be uniquely represented in the I-TBEV group; in the matrix and control groups, instead, only very few genes showed more than 2-fold changes in expression (Figure 1B). A heatmap representation of the differences in expression of all 333 DEGs demonstrates that I-TBEV triggered stronger up- or downregulation of DEGs (compared to the non-stimulated cells) than treatments with HT I-TBEV or matrix (Figure 1C for the average profile per treatment group, Appendix A Figure A1 for sample-specific responses). Interestingly, although HT I-TBEV induced a quantitatively weak response compared to treatment with I-TBEV, the profiles of up- and down-regulated genes were qualitatively similar for the two formulations. This was not the case for the matrix control.

**Figure A2:**
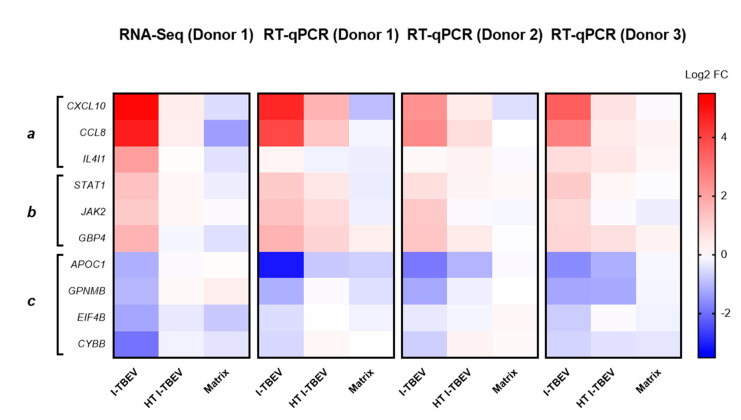
Validation of selected genes identified by RNA-Seq through RT-qPCR. The fold change in PBMCs from 3 donors (including the one used in the RNA-Seq analysis, donor #1) was analyzed in duplicate for each gene and treatment. The clusters represent functions upregulated (**a**,**b**) and downregulated (**c**) by I-TBEV. Results are shown as heatmap (normalized to the expression in the non-treated samples, set as FC = 1), and compared to the RNA sequencing data.

**Figure A3:**
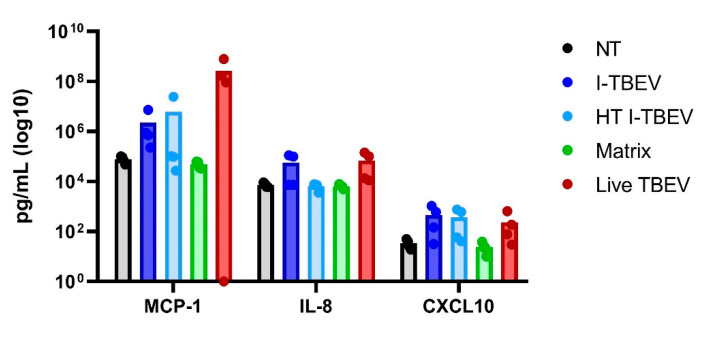
Cytokine production in PBMCs stimulated with conforming, non-conforming I-TBEV and live TBEV. PBMCs from 4 donors were stimulated with the indicated compounds for 24 h, after which the supernatants were collected and the cytokine concentration assessed by Cytometric Bead Assay. Bars represent the average value and dots the individual value per donor.

Three main gene clusters were identified, and selected genes within each cluster were chosen for validation by RT-qPCR. Fold change in gene expression was assessed in three donors (Appendix A Figure A2). The RT-qPCR data for the three donors showed a comparable I-TBEV-induced regulation of the selected genes (with expected donor-dependent differences in the extent of responses) and confirmed the upregulation for clusters *a* and *b* and the downregulation for cluster *c* observed in the RNA sequencing analysis. We also assessed, in PBMCs from four additional donors, the cellular responses at the protein level after 24 h treatment with I-TBEV, HT I-TBEV, matrix and in untreated cells (Appendix A Figure A3). While not statistically significant, a trend became apparent in which incubation of cells with I-TBEV resulted in increased MCP-1, IL-8 and CXCL10 production compared to non-stimulated and matrix-stimulated cells. HT I-TBEV treatment resulted in responses mostly below those to I-TBEV, with, however, donor-dependent variations.

To identify the biological pathways and processes associated with I-TBEV stimulation, we performed functional enrichment analysis of the identified gene clusters using the Database for Annotation, Visualization and Integrated Discovery (DAVID) [23]. The top fifteen hits for each cluster are shown in Figure 1D.

This analysis revealed that, within the clusters *a* and *b* (comprising DEGs strongly and mildly upregulated upon I-TBEV treatment, respectively), the most over-represented functions and pathways were *Defense response to virus*, *Type I interferon signaling pathway*, *Interferon gamma-mediated signaling pathway*, and *Innate immune response*. With a lower but still significant *p*-value, we found activation of the KEGG pathway *RIG-I-like receptor signaling pathway* in cluster *a*, that comprised differentially expressed genes such as *RIG-I*, *MDA5*, *LGP2* and *IRF7*. In cluster *b*, the *JAK-STAT cascade* was identified as upregulated, with DEGs such as *JAK2*, *STAT1*, *STAT2*, and *SOCS*.

The genes in cluster *c*, downregulated in PBMCs treated with I-TBEV, were enriched for processes associated with the *Extracellular exosome*, *Extracellular space* and *Extracellular region*. Additionally, many DEGs in this cluster were enriched for functions related to lipid metabolism, with GO terms such as *Long-chain fatty acid transport*, *Fatty acid binding*, *Lipid metabolic process* and *Lipid catabolic process*. Interestingly, the function *Inflammatory response* was found enriched for both cluster *b* and *c*: selected molecules involved with chemokine, cytokine and interleukin signaling were identified as upregulated (in cluster *b*) or downregulated (in cluster *c*) following treatment with I-TBEV. Thus, while activation of the IFN pathway was identified as an unequivocal signature induced by the main component of the TBE vaccine, genes associated with the inflammatory response were regulated in a highly selective way.

**Figure A4:**
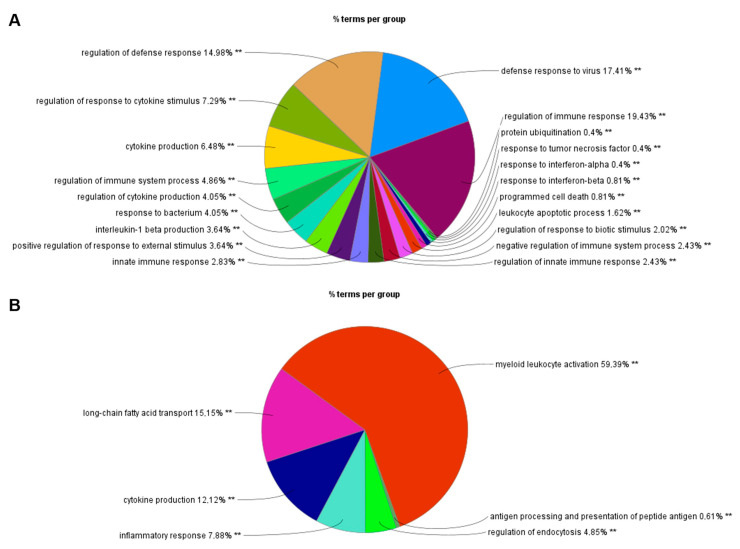
Functional analysis of DEGs identified by RNA-Seq on Cytoscape using the plug-in ClueGO. Functions of DEGs upregulated (**A**) and downregulated (**B**) by I-TBEV treatment. ** indicates a *p*-value < 0.01.

**Figure A5:**
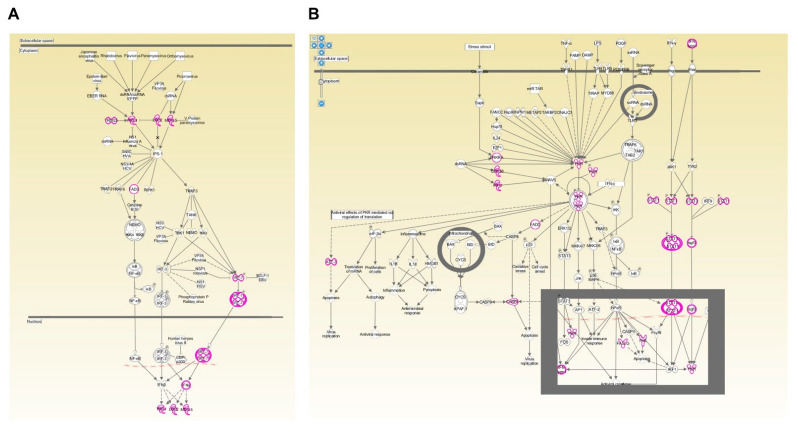
Canonical pathways identified by functional analysis on IPA. Two canonical pathways associated with DEGs upregulated upon I-TBEV treatment are shown: “Role of RLRs in antiviral innate immunity” (**A**) and “Role of PKR in interferon induction and antiviral response” (**B**). DEGs identified by the RNA-Seq are highlighted.

**Figure A6:**
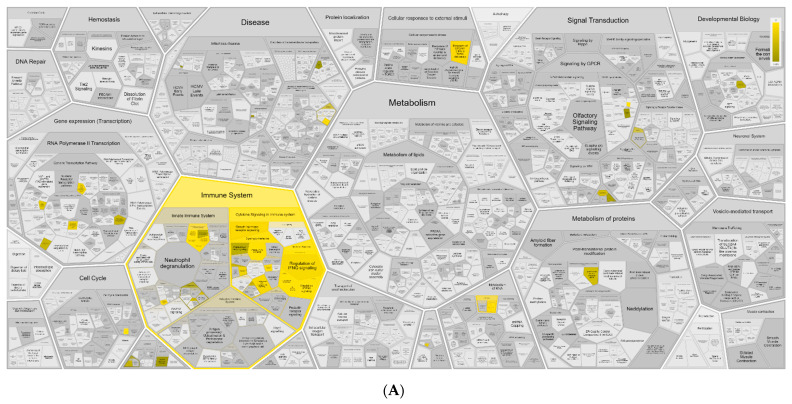

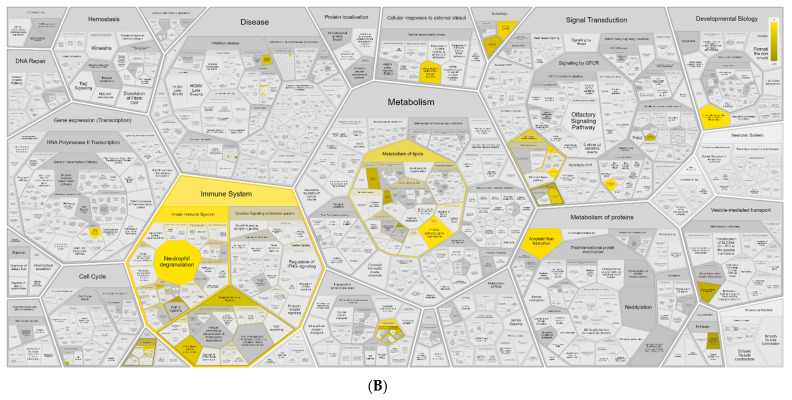
Functional analysis of DEGs identified by RNA-Seq on Reactome. Functions of DEGs upregulated (**A**) and downregulated (**B**) by I-TBEV treatment.

Additional analysis performed using other enrichment tools, such as Cytoscape, Ingenuity Pathway Analysis and Reactome, identified similar pathways associated with the stimulation of PBMCs with I-TBEV, in particular its induction of IFN responses, upregulation of RLRs and downregulation of genes associated with lipid metabolism and selected inflammatory responses (Appendix A Figure A4, Figure A5 and Figure A6). Overall, functional enrichment of differentially expressed genes in PBMCs treated with I-TBEV showed an interferon-dominated immune profile, and indicated a role for cytosolic pattern recognition receptors belonging to the RIG-I-like family.

### 3.2. PBMCs Treated with Live and Inactivated TBEV Share Similar Transcriptional Profiles

**Figure A7:**
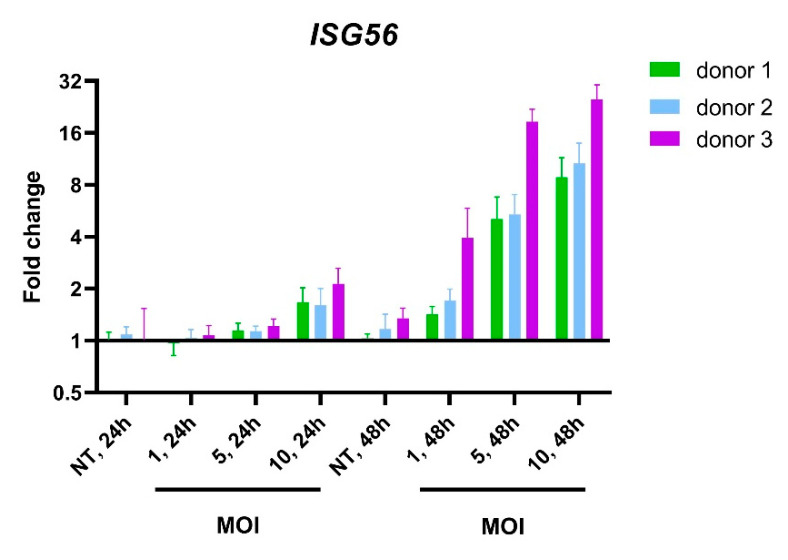
Upregulation of *ISG56* in PBMCs incubated with live TBEV. Changes in gene expression following 24 or 48 h incubation with different multiplicities of infection (MOIs) of live virus were analyzed by RT-qPCR in PBMCs from 3 healthy donors.

To compare the innate immune signature of cells treated with the inactivated TBEV to that of cells incubated with the live virus, a second RNA-Seq analysis was performed on the data from the control, I-TBEV- and live TBEV-treated groups (Analysis 2). The transcriptional profile for the live virus group was assessed with cells incubated at a multiplicity of infection of 10 for 48 h, since experiments indicated that an infection of 24 h induced minimal changes in the expression of IFN stimulated genes (ISGs) (Appendix A Figure A7) [17].

**Figure 2:**
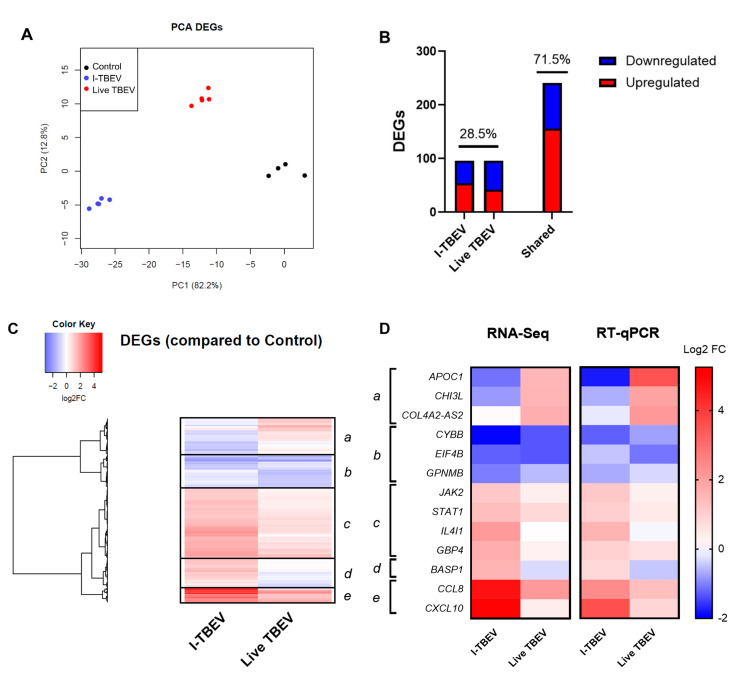
Transcriptional analysis of PBMCs stimulated with I-TBEV and live TBEV (Analysis 2). PBMCs from a healthy donor were stimulated for 24 h with inactivated TBEV (I-TBEV) at a concentration of 0.24 µg/mL or 48 h with live TBEV at an MOI of 10. After treatment, the cells were lysed and processed for RNA sequencing. (**A**) Principal component analysis based on DEGs. (**B**) Representation of DEGs with distinct or overlapping change in expression in the I-TBEV and live TBEV treatment groups as compared to the control. (**C**) Heatmap showing the FC of differentially expressed genes (DEGs) in the two treatment groups, normalized to the control group (set as FC = 1) and hierarchically clustered. (**D**) Validation of RNA-Seq results by RT- qPCR. The fold change in PBMCs was analyzed in duplicate for each gene and treatment, and was averaged for all 3 donors. Results are shown as heatmap and compared to the RNA sequencing data.

After discarding unexpressed and non-differentially expressed genes, 337 DEGs were obtained from the analysis of the control group, I-TBEV- and TBEV-treated cells. The PCA for differentially expressed genes shows distinctive clustering of the three treatment groups (Figure 2A). Stimulation with I-TBEV and live virus induced partially overlapping expression signatures (Figure 2B): the DEGs showing similar regulation in both treatment groups accounted for 71.5% of the total, while 28.5% of the genes showed opposite transcriptional signatures in I-TBEV- and live virus-treated cells. The heatmap representation in Figure 2C displays the DEGs organized by hierarchical clustering, which identifies 5 major clusters: 3 include genes similarly up- (clusters *c* and *e*) or downregulated (cluster *b*) in both treatment groups, while 2 include DEGs that are downregulated in I-TBEV-treated cells and upregulated in live TBEV-treated cells (cluster *a*), or vice versa (cluster *d*). 

To validate these findings, and confirm that the differential profile induced by the live virus was not resulting from the extended incubation time, the expression of selected genes within each cluster was validated by RT-qPCR in PBMCs (from the aforementioned 3 donors, including the donor whose cells were analyzed by RNA-Seq) treated with I-TBEV or live TBEV for 24 h. The changes in gene expression, averaged for the 3 donors, are shown and compared to the results from the RNA sequencing (Figure 2D). The expression signature induced by the virus was confirmed, as the genes in each cluster showed a similar up- or downregulation as observed in the NGS results.

Again, we evaluated the cytokine production in PBMCs from 4 donors in response to I-TBEV and live TBEV stimulation (Appendix A Figure A3). On average, production of IL-8 and CXCL10 was increased with both treatments, while the protein level of MCP-1 appeared to be higher following incubation with the live virus (compared to inactivated TBEV) in all but one donor.

**Figure 3:**
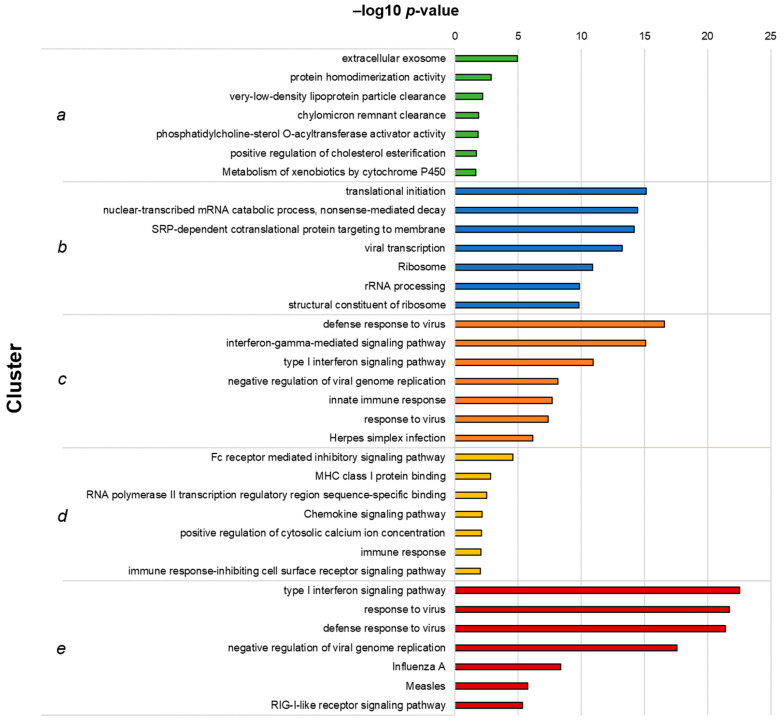
Top 7 GO terms and KEGG pathways identified in each cluster from Analysis 2. Hierarchical clusters (as presented in Figure 3C) were analyzed for functional enrichment. The clusters represent functions similarly up- (clusters (**c**,**e**)) or downregulated (**b**) by live TBEV and I-TBEV, downregulated in I-TBEV-treated cells and upregulated in live TBEV-treated cells (**a**), or vice versa (**d**). The bars show the –log10 of the raw *p*-value.

Functional enrichment analysis of the clusters was performed as previously described, and the top 7 hits for each cluster are shown in Figure 3. Cluster *a*, which included genes downregulated following I-TBEV treatment and upregulated upon incubation with the live virus, comprises functions related to lipid metabolism, a process already identified in Analysis 1. Cluster *b*, including DEGs downregulated in both sample groups, shows enrichment of terms involved with RNA metabolism. Clusters *c* and *e*, both including genes upregulated in I-TBEV- and live virus-treated cells, show an over-representation of pathways related to the antiviral and IFN response and RLR signaling. Cluster *d*, comprising DEGs upregulated upon treatment with I-TBEV and downregulated by the live virus incubation, includes functions involved in the immune response.

In summary, the analysis of DEGs identified in inactivated and live virus-treated PBMCs showed that the transcriptional profiles induced by the two treatments were mostly similar, with the exception of selected immune pathways (induced by the inactivated virus only) and of functions involved in the lipid metabolism (upregulated only upon incubation with the live virus). These differences may be ascribed to the intrinsic nature of the replicating virus, which acts to suppress certain immune responses [27] and to induce intracellular membrane rearrangements [28,29].

### 3.3. Inhibition of RLRs, But Not of TLRs, Reduces I-TBEV-Induced Responses

To gain a further insight into the molecular cascade engaged by I-TBEV, we assessed the cellular responses in the presence of inhibitors of downstream molecules in selected PRR signaling pathways.

**Figure 4:**
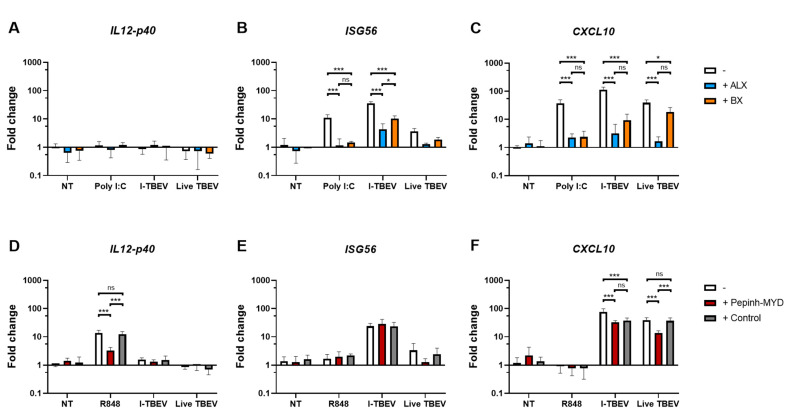
Gene expression levels in PBMCs stimulated with I-TBEV or live TBEV in the presence of inhibitors. PBMCs from 3 healthy donors were pre-incubated with RLR inhibitors (ALX, 50 µg/mL, and BX, 2 µM) for 1 h (**A**–**C**) or with MyD88 inhibitor (Pepinh-MYD, 10 µM) and its peptide control (Control) for 6 h (**D**–**F**). Afterwards, the cells were treated for 24 h with Poly I:C (0.5 μg/mL), R848 (10 μg/mL), I-TBEV (0.24 µg/mL) or live virus (MOI 10). Following stimulation, the cells were lysed and changes in gene expression were analyzed by RT-qPCR. Results are from 3 replicates. Levels of significance: ns: *p* > 0.05; *: *p* ≤ 0.05 and ***: *p* ≤ 0.001. Absence of labels indicates non-significant differences.

**Figure A8:**
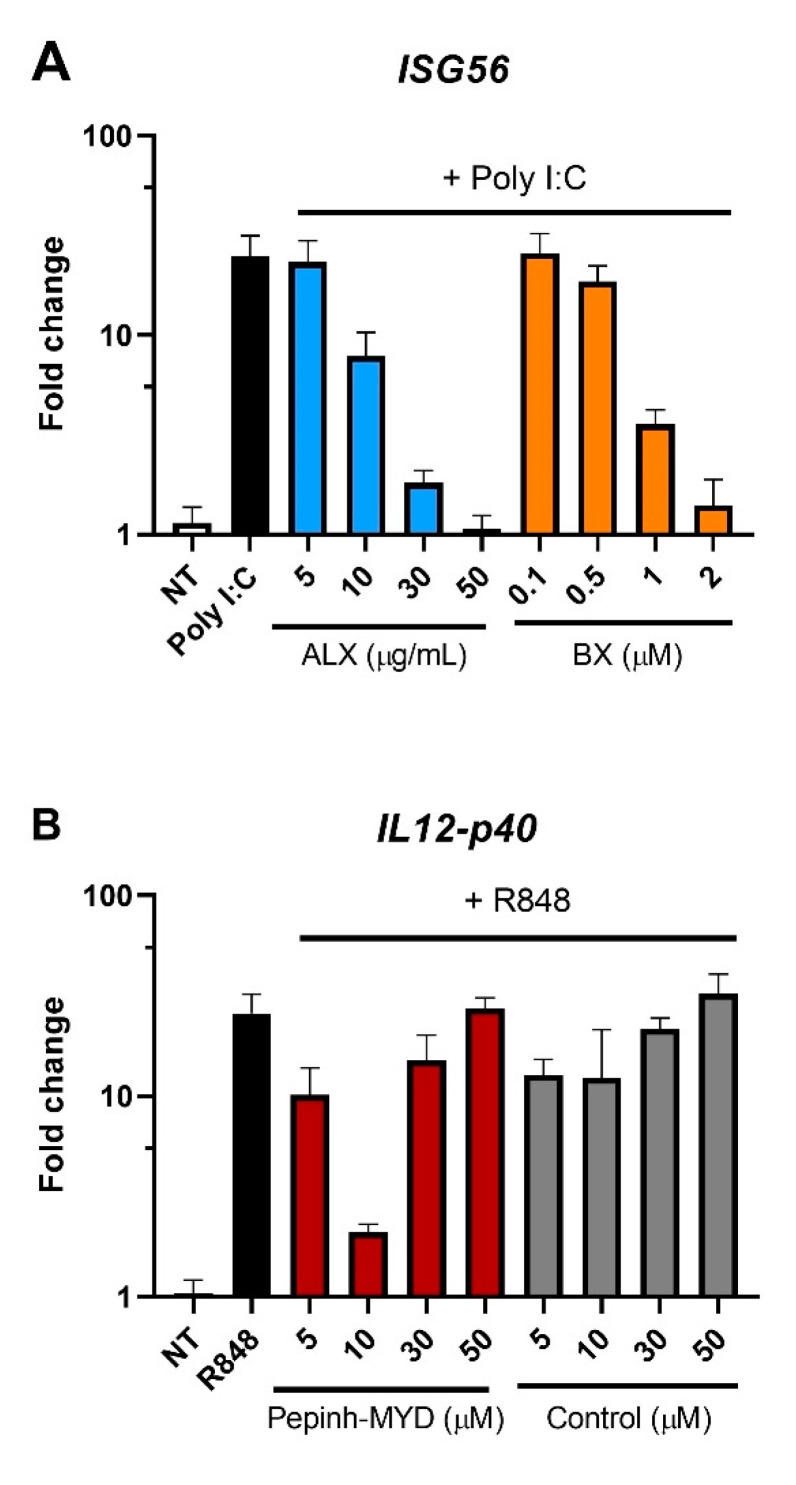
Assessment of the optimal concentration of RLR or MyD88 inhibitors in PBMCs. PBMCs from 3 healthy donors were pre-incubated with RLR inhibitors (ALX and BX) for 1 h (**A**) or with MyD88 inhibitor (Pepinh-MYD) and its peptide control (Control) for 6 h (**B**). Afterwards, the cells were treated for 24 h with Poly I:C (0.5 μg/mL) or R848 (10 μg/mL). Following stimulation, the cells were lysed and changes in gene expression were analyzed by RT-qPCR.

**Figure A9:**
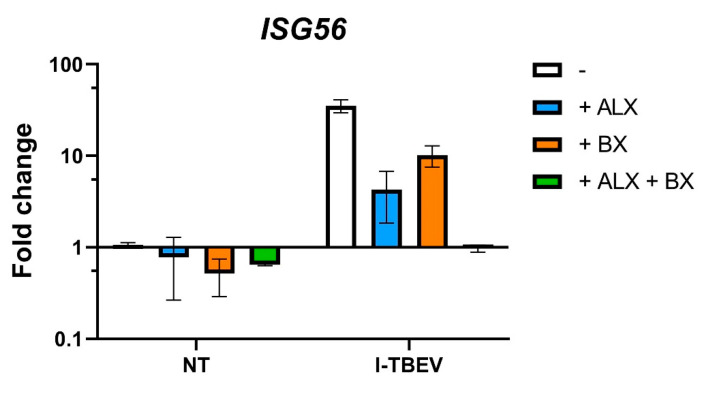
Upregulation of *ISG56* in PBMCs incubated with RLR inhibitors. PBMCs from 3 healthy donors were pre-incubated with RLR inhibitors (ALX at 50 µg/mL and BX at 2 µM) for 1 h. Afterwards, the cells were treated for 24 h with I-TBEV (0.24 µg/mL), or left untreated. Following stimulation, the cells were lysed and changes in gene expression were analyzed by RT-qPCR.

Given the suggestion from the RNA sequencing data of the involvement of RIG-I-like receptors, we first assessed the expression of biomarkers associated with I-TBEV stimulation upon treatment with Amlexanox (ALX) and BX795 (BX), two specific inhibitors of the noncanonical IkB kinases IKKε and TANK-binding kinase 1 (TBK1) [30,31] downstream of RLRs [32]. By monitoring the expression of *ISG56* in PBMCs from 3 healthy donors upon treatment with the positive control Poly I:C, we established 50 μg/mL to be the optimal concentration of ALX and 2 μM that of BX in terms of inhibitory capacity of the compounds (Appendix A Figure A8A). ALX and BX did not induce off-target effects such as activation of the inflammatory pathway (assessed as *IL12-p40* expression, Figure 4A). The presence of ALX or BX during stimulation with inactivated TBEV resulted in a decreased expression of *ISG56* and *CXCL10*, two I-TBEV-induced biomarkers [17], indicating the involvement of the RIG-I pathway in the transduction of I-TBEV-associated signals (Figure 4B,C). At the conditions used, ALX imposed the strongest reduction, and the reduction was enhanced when both inhibitors were used in combination (Appendix A Figure A9). We also assessed the effect of the two inhibitors on signaling by live TBEV; both ALX and BX significantly decreased the expression of *CXCL10* (Figure 4C) and, albeit not significantly, of *ISG56* (Figure 4B).

Next, we sought to analyze the possible involvement of TLRs, as I-TBEV contains intact viral RNA reported to stimulate plasmacytoid dendritic cells through TLR7/8 activation [33]. To do this, we used a MyD88 inhibitor peptide, Pepinh-MYD, interfering with the transduction of the respective TLR signaling cascade [34]. Using R848 as ligand for TLR7/8 and expression of *IL12-p40* as readout [17,35,36,37], we first established a concentration of 10 μM Pepinh-MYD as capable of significant and specific inhibition of the TLR signaling cascade (Appendix A Figure A8B). When used at this concentration, Pepinh-MYD did not have an effect on the induction of *ISG56* by I-TBEV (Figure 4E). Expression of *CXCL10* was affected by Pepinh-MYD as well as by the peptide control to an equal extent, pointing to an unspecific effect (Figure 4F). Thus, the MyD88 pathway was likely not involved in I-TBEV-evoked responses. In contrast, the upregulation of *ISG56* and *CXCL10* upon incubation with the live virus was affected by Pepinh-MYD in a specific manner, pointing to an involvement of the MyD88 pathway in live TBEV signaling.

### 3.4. TLR and NOD Reporter Cells Do Not Respond to I-TBEV, But Can Be Activated by Live TBEV

To further investigate the involvement of selected PRRs in the sensing of (I-)TBEV, we turned to HEK Blue cells, human cells engineered to express a reporter construct upon ligand binding to various PRRs (individually expressed in each cell line). The cells were incubated for 48 h with increasing concentrations of I-TBEV or its matrix control, and their level of stimulation is reported as a percentage of activation of the cells compared to the response to a fixed amount of TNF-α (set as 100% activation).

**Figure 5:**
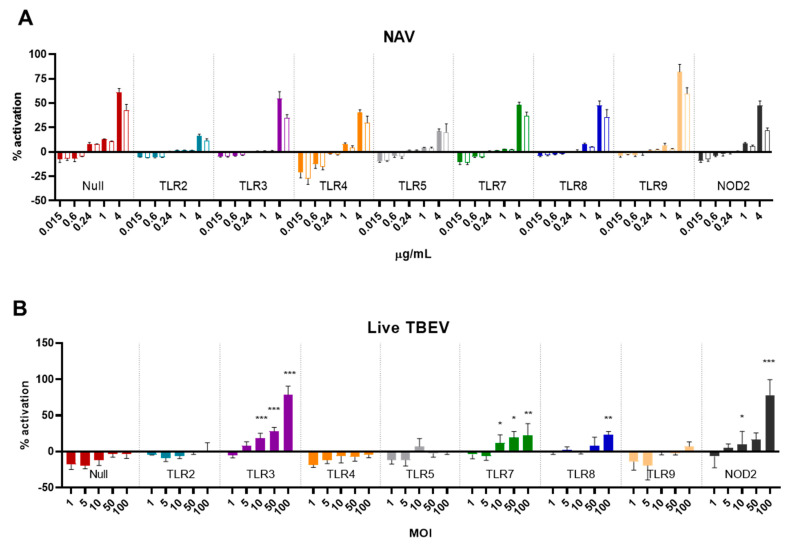
Activation of HEK Blue reporter cells by I-TBEV and live TBEV. (**A**) HEK Blue cells were stimulated with the indicated amounts of I-TBEV for 48 h at different concentrations. Subsequently, supernatants were added to the detection medium for assessment of NF-κB-induced production of the reporter protein. The stimulation of the cells is presented as a percentage of activation relative to the activation achieved with 2.5 μg/mL of TNF-α (set as 100%). Filled bars represent the responses to I-TBEV, empty bars responses to the matrix. (**B**) HEK Blue cells were stimulated with live TBEV virus for 48 h at the indicated multiplicity of infection (MOI), and activation was assessed as described above. Levels of significance: ns: *p* > 0.05; *: *p* ≤ 0.05; **: *p* ≤ 0.01 and ***: *p* ≤ 0.001 (compared to the level of activation in the Null cells stimulated with the same MOI). Results are from 3 replicates.

While the different HEK Blue cell lines responded to their specific TLR and NOD2 ligands, none of the receptors were triggered specifically by I-TBEV (Figure 5A). The production of the reporter protein, found only at the highest concentration of I-TBEV used, was also induced by the same dose of sucrose-containing matrix solution alone, and, more importantly, the activation of the NF-κB pathway was also observed in the parental ‘Null’ cell line lacking all PRRs. Interestingly, when incubated with the live virus, TLR3, 7, 8 and NOD2-expressing cells did show the expression of the reporter protein in a dose-dependent manner (Figure 5B). Thus, while some receptors could be activated by the live TBEV, the inactivated virus was unable to trigger PRR-specific responses in any of the reporter cell lines, corroborating the findings obtained in the frozen-thawed PBMCs.

## 4. Discussion

In this study, we assessed the cellular responses and pathways induced by I-TBEV, the main component of the TBE vaccine, and by the replicating virus in human primary cells and reporter cell lines. Our first aim was to extensively characterize the innate immune signature induced by I-TBEV in our human PBMC platform in comparison to untreated cells, as well as to cells treated with low-quality I-TBEV or live virus. The second aim was to identify the pattern recognition receptors responsible for (I-)TBEV sensing.

Addressing the first aim, we found that I-TBEV induced an interferon-dominated immune profile and the upregulation of selected inflammatory genes. The low-quality formulation, HT I-TBEV, triggered a qualitatively similar expression signature; however, it had much lower response magnitudes. While the sequencing results were obtained in cells derived from only one donor, the same expression pattern emerged in the validation of the results with 2 additional donors, and the findings are in line with previous results on selected I-TBEV-induced responses in primary human immune cells from several donors [17,33]. Indeed, the induction of IFN responses has been identified as a common early signature for several vaccines [38]. Interestingly, selected genes involved in antigen presentation, interleukin signaling and interactions between lymphoid and non-lymphoid cells were underexpressed following treatment of PBMCs with I-TBEV. This downregulation of certain immune functions was for us unexpected, and were in contrast with results from studies on whole inactivated influenza virus, which found some of the same molecules upregulated in primary cells [21,39,40]. Thus, induction of selected inflammatory markers in vitro can be highly pathogen-specific. Nevertheless, the successful activation of an interferon cascade appears to be a general feature of promising vaccine candidates and was shown to correlate with favorable antibody titers [41,42] and T cell responses [11,43] in vivo.

Comparing the immune profiles in cells incubated with live or inactivated TBEV, we observed that the live virus induced a transcriptional signature overlapping in large parts with that induced by I-TBEV, with the exception of replication-related genes distinctly regulated in live virus-treated cells. Similarities in immune responses to live and whole inactivated viruses were previously observed in other studies [39,40], but the signature identified is of course cell- and pathogen-specific. TBEV, as many other (flavi)viruses, tries to evade the immune system during infection through (1) replication in membrane vesicles hindering the activation of PRRs, (2) inhibition of signaling cascades by non-structural proteins. and (3) impairment of antigen-presenting cell (APC) maturation [44,45,46,47,48]. Downregulation of IFN production, a known TBEV defense mechanism, is restricted to the early stages of infection, and—as was also evident in this study—interferon signaling recovers after 24 h [29,49]. However, suppression of certain functions is still ongoing at this timepoint. Downregulation in the expression of adhesion molecules was previously reported in TBEV-infected cells [50,51]; our results extend this finding also to cells incubated with the inactivated virus. The induction of membrane rearrangements appears instead to be specific for the replicating virus, as in our platform only live TBEV induced upregulation of the cellular pathways involved in lipid metabolism. Overall, given the induction of interferon responses by the inactivated virus at similar or higher levels than those induced by the live virus, I-TBEV stands out as a potent vaccine component.

After having established the distinctive transcriptional profile of (I-)TBEV-stimulated cells, we sought to determine which pattern recognition receptors, once triggered, led to the identified responses. Using inhibitors of downstream factors of PRRs, we demonstrated the involvement of RLRs in I-TBEV sensing in cryopreserved PBMCs. Inhibition of MyD88—an adapter protein downstream of TLRs—did not affect the expression of I-TBEV-induced genes such as *ISG56* and *CXCL10*, while inhibition of TBK1/IKKε—factors downstream of RLRs—halted the signaling cascade.

Activation of RIG-I-like receptors is a predominant mechanism for cellular recognition of flaviviruses [52,53]; expression of RIG-I and MDA5 is enriched in human neural cells following TBEV infection [54], and inhibition of RLR signaling was found to suppress TBEV-induced interferon production [28,46,55]. RIG-I detects uncapped (with an exposed 5′-triphosphate group) single-stranded RNAs (ssRNAs) and, together with MDA5, double stranded RNAs (dsRNAs), which are both produce during viral replication [56]. As such, RLRs should not be triggered by the inactivated virus, since the viral genome is packaged once mature and is capped [57]. However, activation of RIG-I by panhandle RNA (a partially circularized structure) lacking a 5′-PPP moiety has been demonstrated for influenza A virus [58]. Given the presence of cyclization elements in TBEV (and other flaviviruses) RNA [59,60], we hypothesize that, after the uncoating of the (inactivated) virus, such structures can be recognized by RIG-I in the cytosol. 

The role of the viral genome in the immunogenicity of I-TBEV was previously assessed in plasmacytoid dendritic cells (pDCs) derived from freshly isolated PBMCs, which were found to sense I-TBEV through TLR7/8 [33]. The discrepancy with the results described here could be explained by the fact that cryopreserved PBMCs were used in this study. Cryopreservation can alter the relative proportions of APCs [61] and decrease the amount of pDCs [62], or affect cellular responses to TLR agonists [63,64]. Furthermore, the RIG-I pathway is dispensable for IFN production in pDCs, while it is of crucial importance in other DCs and in non-dendritic APCs [49]. A comparison of the responses to I-TBEV in fresh and frozen-thawed PBMCs and in the presence of RLR and TLR inhibitors could confirm that distinct pathways are predominant in different cell subtypes. Overall, the contribution of the viral ssRNA to the activation of APCs appears to be a prominent feature of the TBE vaccine. For influenza, it has been shown that vaccines containing the viral genome—able to activate endosomal ssRNA receptors [65]—induce stronger immune responses than formulations lacking it [21,39,66], presumably through the activation of more diverse molecular pathways. These considerations should therefore be taken into account more widely during vaccine development, as also for TBEV, the presence of the viral genome is shown to provide self-adjuvanting properties.

Our study provides new insights into how I-TBEV activates the innate immune system in vitro. Understanding the mechanism of action through which a virus particle interacts with the immune system can offer several benefits. Firstly, comparison between the live and the inactivated virus could, for example, indicate whether critical viral components are retained during the manufacturing process of inactivated virus vaccines. Secondly, the identification of key pathways might allow the selection or design of cellular platforms which express the relevant components. Such platforms would be particularly suited for assessing the potential of the vaccine in vitro. Thirdly, given that the TBE vaccine is highly effective, knowledge of the innate pathways engaged by this vaccine provides a lead for the design of effective vaccines for other pathogens.

However, it has to be realized that the TBE vaccine contains aluminum hydroxide as an adjuvant, which by itself also affects the innate immune system [67,68,69,70]. Unfortunately, the responses to the final vaccine formulation could not be analyzed in our platform since the alum adjuvant appeared not to be compatible with the viability of PBMCs [17]. Therefore, while some conclusions can be drawn from an I-TBEV-based analysis of the immune signature in human PBMCs, a complete picture of the in vitro responses to the final TBE vaccine can be achieved only once an adjuvant-tolerating platform is found. An additional limitation of the study is that these innate responses were only analyzed in vitro. While several of the genes identified in this study had earlier been described to be involved in the responses to live TBEV in vivo [44,71,72,73], the findings concerning vaccine-specific responses have yet to be confirmed in mouse models and in immune cells from vaccinated individuals.

As the scientific community strives to bring safe and effective SARS-CoV-2 vaccines to the market, the process of vaccine development and assessment needs global attention now more than ever. While vaccine potency and effectiveness can be assessed relatively easily, knowledge of which host pathways should be activated for mounting a sufficient (but not excessive) immune response is not yet solid. This is especially the case with ‘difficult’ vaccines (for rapidly mutating pathogens, as well as for viruses and bacteria with complex interactions with the host’s immune system), where traditional “isolate, inactivate and inject” strategies might be inappropriate for vaccine development. Next generation sequencing techniques have been proposed as tools eventually enabling rational and directed vaccine design [74,75,76]. As such, the present study contributes to the growing evidence of their applicability for understanding the mechanisms of action of vaccines and their components.

## Data Availability

The data presented in this study are available on request from the corresponding author. The data are not publicly available due to restrictions from the funding consortium.

## Appendix A

**Table A1:** Primer list for the genes assessed through RT-qPCR.

Gene	Forward	Reverse	Source
*IL12-p40*	CTGCCCAGAGCAAGATGTGTC	CATTTCTCCAGGGGCATCCG	Own design
*ISG56*	CCTGGAGTACTATGAGCGGGC	TGGGTGCCTAAGGACCTTGTC	Holzinger et al., JVirol (2007)
*CCL-8*	GTTTCTGCAGCGCTTCTGTG	TGGCTGAGCAAGTCCCTGA	Ma et al., Exp Terap Med (2016)
*CXCL-10*	TGAAATTATTCCTGCAAGCCAA	CAGACATCTCTTCTCACCCTTCTTT	Ma et al., Exp Terap Med (2016)
*STAT1*	TGCAAATGCTGTATTCTTCTTTGG	TATGCAGTGCCACGGAAAGC	Zhang et al., Immunol (2009)
*IL4I1*	GCTGAAGAAAGAAGAAACCCACC	CCTAACTGCCACAGAAGGGA	Own design
*CHI3L1*	TGCCCTTGACCGCTCCTCTGTACC	GAGCGTCACATCATTCCACTC	Erdman et al., MalariaJ (2014)
*JAK2*	TTCAGAAGCAGGCAACAGG	TCTGTCATCGTAAGGCAGGC	Warby et al., J Virol (2003)
*APOC1*	TTCTGTCGATCGTCTTGGAA	TCAGCTTATCCAAGGCACTG	Ko et al., ThoracicCan (2014)
*CYBB*	TAGTGGGTCCCATGTTTCTGTATC	ACATCACCACCTCATAGCTGAA	Okura et al., J Clin Immunol (2015)
*EIF4B*	GGCTGATGAAACGGATGACCT	GGTCGATATTGGGTTCCCGA	Nowak et al., EBioMedicine (2019)
*GBP4*	CCGGCCTACAAATGACAAGC	AGCCGCTTTCCAGTGACAAT	Own design
*COL4A2-AS2*	CTCTCAGGTCATGCCCATCC	CTGAGTCCTGTGCACGTCTT	Own design
*GPNMB*	TGCTGACTGTGAGACGAACC	CACCAAGAGGGAGATCACAGT	Own design
*BASP1*	CAACTGGCTCCTCGCTCC	TGAGCTTGCCTCCCATCTTG	Own design
*GAPDH*	AGGGCTGCTTTTAACTCTGGT	CCCCACTTGATTTTGGAGGGA	Abubaker et al., PLOS One (2013)
